# Resident and Faculty Attitudes Toward the Dutch Radiology Progress Test as It Transitions from a Formative to a Summative Measure of Licensure Eligibility

**DOI:** 10.1007/s40670-018-0605-7

**Published:** 2018-08-17

**Authors:** D. R. Rutgers, J. P. J. van Schaik, W. van Lankeren, F. van Raamt, Th. J. ten Cate

**Affiliations:** 1 Department of Radiology, University Medical Center, Utrecht, The Netherlands; 2 Department of Radiology, Erasmus University, Rotterdam, The Netherlands; 3 Department of Radiology, Gelre Hospital, Apeldoorn, The Netherlands; 4 Center for Research and Development of Education, University Medical Center, Utrecht, The Netherlands

**Keywords:** ᅟ

## Abstract

**Background:**

Progress testing, a regularly administered comprehensive test of a complete knowledge domain, usually serves to provide learners feedback and has a formative nature.

**Objective:**

Our study aimed to investigate the acceptability of introducing a summative component in the postgraduate Dutch Radiology Progress Test (DRPT) among residents and program directors in a competency-based training program.

**Methods:**

A 15-item questionnaire with 3 items on acceptability of summative postgraduate knowledge testing, 7 on acceptability of the summative DRPT regulations, 4 on self-reported educational effects, and 1 open comment item was distributed nationally among 349 residents and 81 radiology program directors.

**Results:**

The questionnaire was filled out by 330 residents (95%) and 48 (59%) program directors. Summative postgraduate knowledge testing was regarded as acceptable by both groups, but more so by program directors than residents. The transition toward summative assessment in the DRPT was received neutrally to slightly positively by residents, while program directors regarded it as an improvement and estimated the summative criteria to be lighter and less stressful than did residents. The residents’ self-reported educational effects of summative assessment in the DRPT were limited, whereas program directors expected a greater end-of-training knowledge improvement than residents.

**Conclusions:**

Both residents and program directors support summative postgraduate knowledge testing, although it is more accepted by program directors. Residents receive summative radiological progress testing neutrally to slightly positively, while program directors generally value it more positively than residents. Directors should be aware of these different perspectives when introducing or developing summative progress testing in residency programs.

## Introduction

Competency-based medical education (CBME) flourishes in many countries [1]. Through CBME, medical trainees learn the indispensable competences to practice medicine later in professional life [2, 3]. Assessment is challenging in CBME, as it should both stimulate learning and ensure trainees’ readiness to progress [4]. Fail decisions may warrant longer education, or early pass decisions shorter training, which is referred to as time-variable training in CBME [5]. To assess competences, both workplace-based assessment and knowledge and skill tests may be used. Progress testing is a comprehensive knowledge assessment that is administered multiple times per year to all learners in a given curriculum [6–10]. It has been embraced by many medical schools [11]. In postgraduate medical education, progress testing is less frequently applied and usually has a formative nature [11–14]. However, in the setting of CBME, educators need a summative format to decide whether trainees are ready to move on [15]. For postgraduate progress tests, this may ask for a transition from formative to summative formats. In radiological CBME, important competences are radiological knowledge and image interpretation skills. Because they form a set of varying competences that can be simultaneously assessed in a single digital progress test, radiology is an attractive subject for CBME study compared with other medical specialties.

In the definition of good assessment, acceptability and educational effects are important characteristics [16, 17]. Acceptability is the degree to which stakeholders such as learners, educators, and institutions support the assessment method and its scores [16]. Educational effects refer to the impact of assessment on current and future education [17]. From undergraduate education, it is known that progress testing has a positive learning effect by discouraging binge learning and promoting long-term knowledge retention [8, 18]. As progress testing is still relatively uncommon in residency [11–14], and summative progress testing even more so, little is known about the acceptability and educational effects of progress testing in a postgraduate setting. Dijksterhuis et al. found good acceptability but limited self-reported educational effects in formative progress testing in obstetrics and gynecology residents [12]. For summative postgraduate progress testing, acceptability and educational effects have not been described. To start to filling up this gap, we conducted the present study in which we surveyed Dutch residents and program directors in a competency-based radiology training program. This program included a progress testing format that transitioned from a formative to a summative nature.

### Educational Setting

Radiology residency in the Netherlands comprises a 5-year competency-based training program. Throughout the training program, radiology residents are formatively and summatively assessed in numerous workplace observations and written examinations, including the Dutch Radiology Progress Test (DRPT). Radiology residency in the Netherlands does not include a board exam, but graduation from the training program has to be reinforced by the national registration committee for medical specialists in order for the resident to register as a radiologist. The DRPT has been a formative assessment tool in the training program since 2003. It is a semi-annual comprehensive radiological knowledge test with required participation during the complete 5-year residency period [19], resulting in a total of ten tests during residency. Previous studies have shown more than acceptable reliability of the DRPT as a formative assessment tool and have provided support for test validity by demonstrating increase in scores on radiological knowledge and skills in the first 3 years of residency [19–21]. In July 2014, the DRPT was adapted to include a summative (pass) requirement before completion of residency to enhance learning and to meet the need for accountability [17]. The summative regulations only applied to trainees entering residency from July 2014 onward (“summative DRPT group” in the present study). For those who had already started residency before, the DRPT remained formative in all training years (“formative DRPT group”). This transition provided a unique opportunity to study the acceptability of summative testing.

### Summative Regulations in the Dutch Radiology Progress Test

**Fig. 1:**
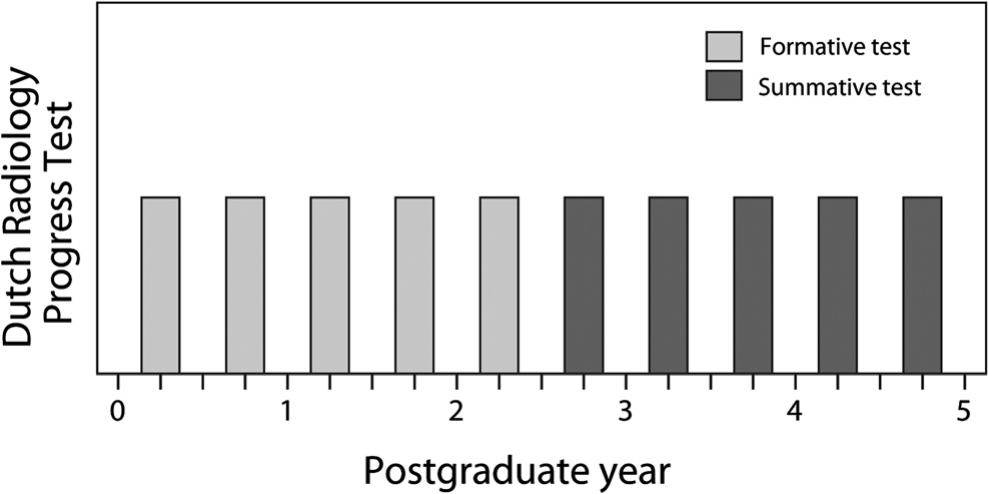
Overview of required individual tests of the Dutch Radiology Progress Test (DRPT) during the 5-year training program of radiology residency in the Netherlands, after the introduction of summative regulations. Residents must obtain a pass score for at least three of the five individual tests that are taken in postgraduate years 2.5 to 5. Tests in the first 2.5 years of training are formative and do not contribute to summative decisions

The DRPT’s pass/fail criterion was defined as follows: residents must obtain a pass score for at least three of the five individual tests that are taken in postgraduate years (PGYs) 2.5 to 5. Tests in the first 2.5 years of training remain all formative (Fig. 1). Residents at risk of failing to reach three sufficient test scores by the end of PGY 5 are obliged to take (and pass) the examination for the European Diploma in Radiology (EDiR) of the European Society of Radiology before completion of residency, as an additional opportunity to demonstrate an adequate radiological knowledge level. If a resident does not pass either of these summative criteria, registration as a radiologist in the Dutch medical register is postponed. Residents are allowed to re-sit for examinations until a pass score is achieved, complying with the competency-based nature of the training program. They can re-sit examinations as often as necessary, at an interval with which these examinations are normally offered. No extra examinations are organized. Following the DRPT’s pass/fail criterion, the April 2017 DRPT was the first test that actually counted summatively for individual residents who had entered residency from July 2014 onward.

### Study Purpose

Our purpose was to study the acceptability and self-reported educational effects of introducing a summative component in postgraduate progress testing within a competency-based radiology training program.

## Materials and Methods

### Research Design

We conducted a cross-sectional, descriptive study in Dutch radiology residents and program directors. We surveyed them with a questionnaire to assess acceptability and self-reported educational effects of transitioning from formative to summative progress testing.

### Questionnaire

We performed a PubMed search for relevant questionnaires with the terms “postgraduate education,” “progress test,” and “assessment,” yielding one questionnaire from Dijksterhuis et al. on acceptability and educational effects of formatively used postgraduate progress testing [12]. Based on the three-part structure of their questionnaire (general acceptance, acceptability of specific test content, and educational impact), we designed a new digital questionnaire with items on three topics (general acceptability of summative progress testing, specific acceptability of the DRPT regulations, and self-reported educational effects) that was tailored to the educational setting of our study, making use of feedback from three radiologists, one medical education expert, and three radiology residents (PGY 2, 4, and 5) on preliminary drafts.

The questionnaire consisted of three items on acceptability of summative postgraduate knowledge testing, seven on acceptability of the summative DRPT regulations, four on self-reported educational effects, and one open comment item. Ten items were applicable to both residents and program directors, and five to residents only. As response formats, we used Likert scales (*n* = 11), single-best-answer multiple choice (*n* = 2), and free response (*n* = 2). Items with Likert scales mostly (*n* = 6) included a 5-point scale, typically ranging from “no(t) …” to “very ….” The other Likert items had a 7-point (*n* = 4) or 9-point (*n* = 1) scale with “neutral” or a “neutral”-like answer option in the center of the scale. We chose for these larger scales because in these items, we were interested in the variety of potential responses on both sides of neutral, for which we found a 5-point scale less suitable.

### Participants

We asked all residents (*n* = 349) who participated in the April 2017 DRPT to complete the questionnaire anonymously in the same session as the progress test, but unaware of their final test scores. These residents comprised 92% of all 380 Dutch radiology residents at the time (31 residents were given dispensation from participation). On the same day, we asked all Dutch radiology program directors (*n* = 81) by email to complete the questionnaire anonymously. A reminder was sent 3 weeks later. We encouraged residents and program directors to respond to all questionnaire items, but we left them the possibility to not respond to items.

### Statistical Analysis

**Fig. 2:**
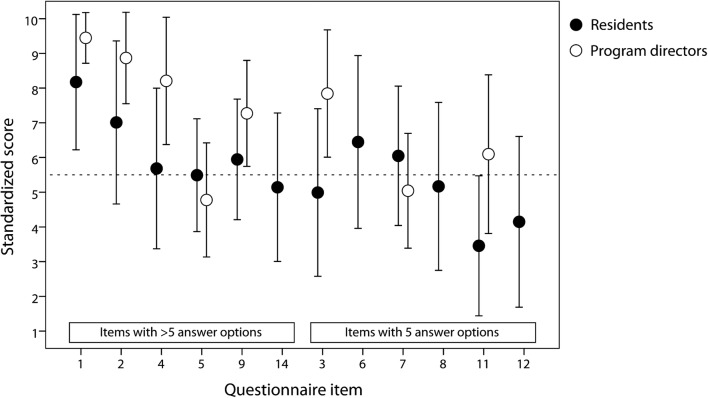
Visual comparison of responses on questionnaire items, extrapolated to a standardized score scale running from 1 to 10. Dots indicate mean and bars standard deviation. The dotted line represents a 5.5 score and is defined as the center score: for items with more than 5 answer options, 5.5 corresponds to the central “neutral”-like answer option in Likert scale items (items 1, 2, 4, 5, and 14) or to the center of the scale in the item about DRPT grading (item 9). For items with 5 answer options (items 3, 6, 7, 8, 11, and 12; all Likert scale items), 5.5 corresponds to answer option “3”. Items 14, 6, 8, and 12 do not apply to program directors. The items on time interval between DRPT passing and specialist registration (item 10) and on DRPT preparation hours (item 13) are not included in the figure because they had no relevant counterpart among the other items for visual comparison

We used Student’s *t* test to analyze differences in training years between the summative and formative DRPT group of residents. Also, we used this test to analyze differences in questionnaire item responses between residents and program directors as well as between the resident subgroups, since parametric statistics are considered a robust and appropriate approach for items with interval response scales [22]. Differences in proportions of absent responses were analyzed with the chi-square test or Fisher’s exact test. For visual comparison between the various questionnaire item responses, we extrapolated the item scores to a standardized score scale ranging from 1 to 10 and summarized scores in a single figure (Fig. 2). In this standardized scale, we defined 5.5 as the center score: for items with more than 5 answer options, 5.5 corresponded to the central “neutral”-like answer option in Likert scale items (items 1, 2, 4, 5 and 14) or to the center of the scale in the item about DRPT grading (item 9). For items with 5 answer options (items 3, 6, 7, 8, 11, and 12; all Likert scale items), 5.5 corresponded to answer option “3”. In all items, the lowest answer option was extrapolated to 1 in the standardized score scale and the highest option to 10. The items on time interval between DRPT passing and specialist registration (item 10) and on DRPT preparation hours (item 13) were not included in the figure because we agreed that they had no relevant counterpart among the other questionnaire items for mutual comparison. A *p* value < 0.05 was considered statistically significant.

### Institutional Review Board Approval

The ethical review board of the Netherlands Association for Medical Education approved conduct of this study (dossier number 927).

## Results

The questionnaire was filled out by 378 respondents, including 330 residents and 48 program directors (response rate 95% and 59%, respectively). All but 2 questionnaire items (item 9 and the open comment item) were answered by at least 371 respondents. The proportion of absent responses did not differ significantly between respondent groups. Participating residents are shown in Table 1. The summative group, in which 8 residents had followed > 2.5 training years, had fewer years of training than the formative group (*p* < 0.001).

**Table 1:** Overview of participating residents

	Residents	
	Summative DRPT group	Formative DRPT group
Number of residents	176	154
Start of training	July 2014 or later	Before July 2014
Number of training years (mean (SD))	1.3 (0.8)	3.7 (0.7)*
DRPTs in training program	Formative in PGY 0–2.5 and summative in PGY 2.5–5	Formative in all PGYs

*DRPT* indicates Dutch Radiology Progress Test; *SD*, standard deviation; *PGY*, postgraduate year

*p < 0.001, versus summative DRPT group, Student’s *t* test

### Acceptability of Summative Postgraduate Knowledge Testing

On average, residents tended to find it fair that knowledge tests are part of medical specialty training in the Netherlands, while they regarded an associated requirement to pass as slightly fair (Table 2). They were inclined to finding knowledge tests moderately important to become a good radiologist. For each of these three items, program directors scored statistically higher than residents (*p* < 0.001), with average responses between “fair” to “very fair” on the first two items and “quite important” on the third item.

**Table 2:** Acceptability of summative postgraduate knowledge testing in non-standardized scores

Questionnaire item		Residents			Program directors (*n* = 48)	Student’s *t* test *p* value (95% CI of the difference)	
Text	Scale	All (*n* = 330)	Summative DRPT group (*n* = 176)	Formative DRPT group (*n* = 154)		Summative versus formative	All residents versus program directors
1. How fair do you find it that knowledge tests are part of medical specialty training in the Netherlands?	1–7^a^	5.8 (1.3)	5.7 (1.3)	5.9 (1.3)	6.6 (0.5)	*p* = 0.30 (− 0.4–0.1)	*p* < 0.001 (− 1.0–− 0.6)
2. How fair do you find it if knowledge tests are required to pass in a medical specialty training?	1–7^a^	5.0 (1.6)	4.9 (1.6)	5.2 (1.6)	6.2 (0.9)	*p* = 0.07 (− 0.7–0.0)	*p* < 0.001 (− 1.5–− 0.9)
3. How important do you find knowledge tests in your own/in your residents’ training program to become a good radiologist?	1–5^b^	2.8 (1.1)	2.8 (1.0)	2.8 (1.1)	4.0 (0.8)	*p* = 0.74 (− 0.3–0.2)	*p* < 0.001 (− 1.5–− 1.0)

Results for residents and program directors are given as mean with standard deviation in parentheses. *CI* indicates confidence interval; *DRPT*, Dutch Radiology Progress Test

^a^Scale items 1 and 2: 1. Very unfair; 2. Unfair; 3. Slightly unfair; 4. Neutral; 5. Slightly fair; 6. Fair; 7. Very fair

^b^Scale item 3: 1. Not important; 2. Slightly important; 3. Moderately important; 4. Quite important; 5. Very important

### Acceptability of the Summative Regulations of the DRPT

The introduction of the summative DRPT did not evoke a clear opinion among the residents (Table 3), while program directors tended to find it an improvement (*p* < 0.001). Residents responded neutrally to the detailed summative DRPT criterion, while program directors tended to find the criterion slightly light (*p* = 0.005). Compared with the formative group, the summative group found the criterion significantly harder (*p* = 0.001) and was less convinced about the ability to meet it (*p* = 0.001). Program directors estimated the summative DRPT as less stressful than residents (*p* = 0.001). On a scale of 1–10, residents generally graded the summative DRPT regulation slightly positively (average grade 5.9), whereas program directors (average grade 7.3) appraised it significantly higher than residents (*p* < 0.001). All groups found that passing a summative DRPT should happen not longer than approximately 1.5 to 2 years before registration as a radiologist.

**Table 3:** Acceptability of summative regulations of the Dutch Radiology Progress Test in non-standardized scores

Questionnaire item		Residents			Program directors (*n* = 48)	Student’s *t* test *p* value (95% CI of the difference)	
Text	Scale	All (*n* = 330)	Summative DRPT group (*n* = 176)	Formative DRPT group (*n* = 154)		Summative versus formative	All residents versus program directors
4. To what extent do you find the introduction of summative assessment in the DRPT an improvement in the radiology training program?	1–7^a^	4.1 (1.5)	4.0 (1.4)	4.3 (1.6)	5.8 (1.2)	*p* = 0.09 (− 0.6–0.0)	*p* < 0.001 (− 2.1–− 1.3)
5. The summative DRPT criterion is: “to obtain a sufficient score in at least 3 of the 5 DRPTs that are taken between year 2.5 and 5 of the 5-year training program”. How light/hard do you find this criterion?	1–9^b^	5.0 (1.4)	5.2 (1.3)	4.7 (1.5)	4.3 (1.5)	*p* = 0.001 (0.2–0.8)	*p* = 0.005 (0.2–1.1)
6. How convinced are you that you can meet the summative DRPT criterion?	1–5^c^	3.4 (1.1)	3.2 (1.0)	3.6 (1.1)	n.a.	*p* = 0.001 (− 0.6–− 0.2)	n.a.
7. How much stress will the summative DRPT give to most residents?	1–5^d^	3.2 (0.9)	3.3 (0.9)	3.2 (0.9)	2.8 (0.7)	*p* = 0.14 (− 0.1–0.3)	p = 0.001 (0.2–0.7)
8. How much stress does the summative DRPT give to you?	1–5^d^	2.9 (1.1)	2.9 (1.0)	2.8 (1.1)	n.a.	*p* = 0.28 (− 0.1–0.4)	n.a.
9. Give a general grade, from 1 (worse) to 10 (best), for the summative DRPT regulations.^e^	1–10	5.9 (1.8) (*n* = 304)	5.8 (1.7) (*n* = 164)	6.1 (1.8) (*n* = 140)	7.3 (1.6) (*n* = 47)	*p* = 0.21 (− 0.6–0.1)	*p* < 0.001 (− 1.9–− 0.9)
10. In your opinion, how many years may maximally go by between passing a summative DRPT criterion as a resident, and actual registration as a radiologist?	1–7^f^	3.9 (2.0)	3.9 (2.0)	3.9 (2.0)	4.2 (2.1)	*p* = 0.73 (− 0.4–0.5)	*p* = 0.43 (− 1.0–0.4)

Results for residents and program directors are given as mean with standard deviation in parentheses. *CI* indicates confidence interval; *DRPT*, Dutch Radiology Progress Test; *n.a.*, not assessed

^a^Scale item 4: 1. Large worsening; 2. Worsening; 3. Slight worsening; 4. Neutral; 5. Slight improvement; 6. Improvement; 7. Large improvement

^b^Scale item 5: 1. Very light; 2. Light; 3. Moderately light; 4. Slightly light; 5. Neutral; 6. Slightly hard; 7. Moderately hard; 8. Hard; 9. Very hard

^c^Scale item 6: 1. Not convinced; 2. Slightly convinced; 3. Moderately convinced; 4. Quite convinced; 5. Very convinced

^d^Scale items 7 and 8: 1. No stress; 2. Slight amount of stress; 3. Moderate amount of stress; 4. Quite an amount of stress; 5. Very much stress

^e^This item was answered by 304 of 330 residents (92%) and 47 of 48 program directors (98%)

^f^Scale item 10: 1. 0–0.5 years (y); 2. 0.5–1 y; 3. 1–1.5 y; 4. 1.5–2 y; 5. 2–2.5 y; 6. 2.5–3 y; 7. > 3 y

### Self-reported Educational Effects

Residents tended to expect the summative DRPT to improve the residents’ end-of-training knowledge level slightly (Table 4), while program directors expected more than moderate improvement (*p* < 0.001). Residents anticipated studying slightly to moderately more for a summative DRPT than for a non-summative test. Compared with the formative group, the summative group reported significantly more preparation for the current DRPT than for the previous one (*p* < 0.001).

**Table 4:** Self-reported educational effects of the summative Dutch Radiology Progress Test in non-standardized scores

Questionnaire item		Residents			Program directors (*n* = 48)	Student’s *t* test *p* value (95% CI of the difference)	
Text	Scale	All (*n* = 330)	Summative DRPT group (n = 176)	Formative DRPT group (n = 154)		Summative versus formative	All residents versus program directors
11. How will the introduction of summative assessment in the DRPT improve the knowledge level that radiology residents will reach at the end of residency?	1–5^a^	2.1 (0.9)	2.0 (0.9)	2.2 (0.9)	3.3 (1.0)	*p* = 0.02 (− 0.4–− 0.0)	*p* < 0.001 (− 1.4–− 0.9)
12. Do you expect to study more for a summative DRPT than if the test would have no summative component?	1–5^b^	2.4 (1.1)	2.3 (1.1)	2.5 (1.1)	n.a.	*p* = 0.25 (− 0.4–0.1)	n.a.
13. Estimate the number of hours that you have spent in the last 2 weeks on focused preparation for the current DRPT.	1–6^c^	3.1 (1.5)	3.0 (1.4)	3.2 (1.5)	n.a.	*p* = 0.39 (− 0.5–0.2)	n.a.
14. How much have you studied for the current DRPT in comparison with your previous DRPT?	1–7^d^	3.8 (1.4)^e^	4.2 (1.4)^e^	3.4 (1.3)	n.a.	*p* < 0.001 (0.5–1.1)	n.a.

Results for residents and program directors are given as mean with standard deviation in parentheses. *CI* indicates confidence interval; *DRPT*, Dutch Radiology Progress Test; *n.a.*, not assessed

^a^Scale item 11: 1. Not improve; 2. Slightly improve; 3. Moderately improve; 4. Quite improve; 5. Largely improve

^b^Scale item 12: 1. (Almost) not more; 2. Slightly more; 3. Moderately more; 4. Quite more; 5. Very much more

^c^Scale item 13: 1. < 1 hour (h); 2. 1–5 h; 3. 5–10 h; 4. 10–15 h; 5. 15–20 h; 6. > 20 h

^d^Scale item 14: 1. Much less; 2. Less; 3. Slightly less; 4. Same as previous; 5. Slightly more; 6. More; 7. Much more; X. Not applicable, this is my first DRPT

^e^44 residents not included as they reported “Not applicable, this is my first DRPT”

### Visual Comparison of Questionnaire Items

Figure 2 shows questionnaire item responses, extrapolated to a standardized score scale running from 1 to 10.

### Open Comments

Open comments were given by 139 (42%) residents and 16 (33%) program directors. Most frequently (approximately one quarter of residents’ and two fifth of program directors’ responses), respondents stated that DRPT test items are too often aimed at factual knowledge that is not relevant for daily clinical practice (“In essence, summative assessment is a good idea, however, test items should then be more representative of daily practice”). Approximately one fifth of residents argued that the DRPT is not representative for clinical performance as a resident or radiologist. Approximately one fifth of program directors responded that progress testing is not suited for summative purposes (“The idea that one should pass, is not in line with the principle of progress testing”).

## Discussion

In this study, we found that both residents and program directors supported summative postgraduate knowledge testing, although it was more accepted by the latter. Program directors had a higher acceptability of summative radiological progress testing than residents who valued it just above neutral. In addition, program directors estimated the amount of related stress to be lower and valued its potential educational effects higher than residents. The varying opinions between residents and program directors may well be related to their different positions in the training program. Program directors carry accountability for the educational program. In their view, having to pass a test may be a better learning stimulus and a stronger proof of competence than merely taking it. Residents on the other hand are the ones who have to pass the test and must potentially face the consequences of failing. Therefore, they may feel more resistance and stress toward summative testing. Stakeholders such as program directors should be aware of these different perspectives when introducing or developing summative progress testing in residency programs. From the residents’ point of view, a program director and administration that are approachable and responsive to the resident’s perspective and concerns will likely contribute to a successful training in radiology [23]. We observed that our summative resident group found the summative DRPT criterion harder and was less convinced about the ability to meet it than the formative group. This may be explained by the fact that the formative group was more experienced than the summative group. Alternatively, since the formative group was free from any summative consequences, it may have been easier for this group to state that summative criteria can be met.

The present study illustrates implementation of summative progress testing in a competency-based postgraduate training program. Our current DRPT regulations stipulate that residents must pass within roughly the last 1.5 PGYs. From the perspective of competency-based education, this time period seems appropriate to make pass/fail decisions on postgraduate radiological knowledge because the second half of residency is generally the time period that the knowledge level of radiology residents matures [21]. In addition, choosing the last 1.5 PGYs as summative time frame fits the average opinion of our respondents that no more than 1.5–2 years should go by between passing a summative DRPT criterion as a resident and the actual registration as a radiologist.

The utility of an assessment method such as the DRPT can be defined as a function of several variables: reliability, validity, cost, acceptability, and educational effects [16]. Previous study of the DRPT has shown more than acceptable reliability over the years and support for its construct validity [19, 20]. The present study adds to this support for acceptability and some support for positive educational effects. The responses to our open comment item make clear that, in line with previous research on postgraduate progress testing [12], residents and program directors welcome daily clinical relevance of test items. Likely, a reduction of highly detailed, factual knowledge items and an increase of practically relevant test items will further increase acceptability of postgraduate progress tests. Acceptability of summative progress testing may be challenged by some program directors’ opinion that progress testing is not suited for summative purposes. Although progress testing is often used formatively, this does not exclude summative components. In fact, progress testing has been deliberately used by others in a summative way to stimulate deep and continuous learning [9].

This study has several limitations. Firstly, the large majority of residents had not (yet) passed a test that actually counted for the summative DRPT criterion. Group perspectives on acceptability and educational effects may change if more residents have taken summative tests. Nevertheless, the present study may provide a good view of residents who are on the verge of a transition toward postgraduate summative assessment. Secondly, we estimated educational effects retrospectively by self-reported questionnaire items. A more precise approach may include prospective study of learning behavior in residents. Thirdly, although we designed our survey with feedback from various stakeholders, further validation of our questionnaire has not yet been performed.

Future study is needed to assess long-term acceptability and educational effects of summative postgraduate progress testing. Our study focused on the period of transitioning from formative to summative progress testing, but acceptability and educational effects should be re-assessed once the summative format has been well established. Also, further study is needed to confirm our findings in other specialties than radiology and to assess validity of the summative DRPT format after the current phase of transition.

## Conclusion

Both residents and program directors support summative postgraduate knowledge testing, although it is more accepted by the latter. Residents receive summative radiological progress testing neutrally to slightly positively, while program directors generally value it more positively than residents. Directors should be aware of these different perspectives when introducing or developing summative progress testing in residency programs.
